# Genome Editing of the* CYP1A1* Locus in iPSCs as a Platform to Map AHR Expression throughout Human Development

**DOI:** 10.1155/2016/2574152

**Published:** 2016-04-11

**Authors:** Brenden W. Smith, Elizabeth A. Stanford, David H. Sherr, George J. Murphy

**Affiliations:** ^1^Section of Hematology and Oncology, Department of Medicine, Boston University School of Medicine, Boston, MA 02118, USA; ^2^Center for Regenerative Medicine (CReM), Boston University and Boston Medical Center, Boston, MA 02118, USA; ^3^Department of Environmental Health, Boston University School of Public Health, Boston, MA 02118, USA

## Abstract

The aryl hydrocarbon receptor (AHR) is a ligand activated transcription factor that increases the expression of detoxifying enzymes upon ligand stimulation. Recent studies now suggest that novel endogenous roles of the AHR exist throughout development. In an effort to create an optimized model system for the study of AHR signaling in several cellular lineages, we have employed a CRISPR/CAS9 genome editing strategy in induced pluripotent stem cells (iPSCs) to incorporate a reporter cassette at the transcription start site of one of its canonical targets, cytochrome P450 1A1 (*CYP1A1*). This cell line faithfully reports on* CYP1A1* expression, with luciferase levels as its functional readout, when treated with an endogenous AHR ligand (FICZ) at escalating doses. iPSC-derived fibroblast-like cells respond to acute exposure to environmental and endogenous AHR ligands, and iPSC-derived hepatocytes increase* CYP1A1* in a similar manner to primary hepatocytes. This cell line is an important innovation that can be used to map AHR activity in discrete cellular subsets throughout developmental ontogeny. As further endogenous ligands are proposed, this line can be used to screen for safety and efficacy and can report on the ability of small molecules to regulate critical cellular processes by modulating the activity of the AHR.

## 1. Introduction

The aryl hydrocarbon receptor (AHR) has been studied for decades for its role in environmental toxin induced carcinogenesis [1, 2]. A member of the Per/ARNT/SIM (PAS) family of basic helix-loop-helix (bHLH) transcription factors, the AHR is activated by small-molecule ligands that cause it to be disassociated from a cytoplasmic chaperone complex and translocated into the nucleus [3]. Upon nuclear translocation, the AHR dimerizes with the Aryl Hydrocarbon Receptor Nuclear Translocator (ARNT) and subsequently binds to conserved AHR response elements (AHREs) within the genome [4]. Through this pathway, the AHR affects the expression of multiple gene targets that contain AHREs in proximal regulatory regions [5, 6]. Classic examples of AHR ligands include 2,3,7,8-tetrachlorodibenzodioxin (TCDD) [7], polycyclic aromatic hydrocarbons (PAHs), and polychlorinated biphenyls (PCBs) [8]. As part of an adaptive response to the presence of these carcinogens, the AHR significantly increases transcription of cytochrome p450 (CYP450) enzymes, specifically* CYP1A1* [9] and* CYP1B1* [10], that will contribute to the metabolism of these compounds into both toxic and nontoxic intermediates [11]. Concomitant with CYP450 activation, the AHR contributes to its own negative regulation by promoting transcription of the AHR Repressor (AHRR), a competitive inhibitor that prevents dimerization of the AHR:ARNT complex, causing free AHR molecules to be exported into the cytoplasm and subsequently degraded [12].

In the last 10 years, there has been a major paradigm shift following the demonstration that the AHR plays important physiological roles in the absence of environmental ligands [13]. Multiple studies suggest the AHR pathway is important in the development and function of the cardiovascular system [14–17] in the* ahr* knockout mouse without the requirement for experimental ligand exposure. Indeed, it is this same model organism that displays varied and diverse developmental phenotypes including, but not limited to, reduced liver size, increased portal tract fibrosis [18], decreased fertility [19, 20], a suspected resistance to neurotoxicity [21], and an impairment in the lymphocyte compartment [22]. AHR signaling has since been implicated in multiple aspects of human developmental ontogeny. Recent studies suggest that the AHR plays a critical role in human hematopoietic stem cell (HSC) differentiation [23], substantiating murine studies that show* in vivo* AHR modulation resulting in disruption of HSC growth, senescence, and migration [24]. Our own work reveals the AHR as a modulator of erythroid and megakaryocyte specification from a common bipotent progenitor [25], incorporating an endogenous ligand of the AHR (6-formylindolo(3,2-b)carbazole or FICZ) to produce this result. Observed roles of the AHR in immunity and inflammation [26] as well as the discovery of novel endogenous AHR agonists [13] add to the overwhelming evidence that AHR signaling is endogenously regulated and crucially important throughout development. Compounded with evidence of regulatory cross talk with VEGF and TGF-*β* pathways [27, 28], these studies seem to suggest that multiple roles of AHR signaling have yet to be discovered.

The advent of cellular reprogramming and genome editing has provided platforms to study signaling pathways in diverse and novel ways. Since their discovery in 2006 [29, 30], induced pluripotent stem cells (iPSCs) have been shown to functionally emulate embryonic stem cells (ESCs) [31, 32] by having the capacity to differentiate into all three germ layers of the developing embryo [29, 30]. iPSCs have been specified to multiple cellular lineages, including those derived from endoderm (liver [33–36], pancreas [37–39], and lung [40–42]), mesoderm (hematopoietic cells [25, 43–48], heart [49–51], and kidney [52]), and ectoderm (neurons [53]). The flexibility of an iPSC-based system allows for the study of multiple tissue types. In this way, iPSCs stand to revolutionize the way we study human development, model disease, and eventually treat patients. Additionally, genome editing strategies have been widely used to create genetic knockouts, repair disease-causing mutations, and integrate reporter constructs [54]. Clustered regulatory interspersed short palindromic repeats (CRISPRs) have been identified as an element of bacterial adaptive immunity by which foreign DNA of invading species is incorporated into the host genome and subsequently used as a template upon which CRISPR associated (CAS) endonucleases bind and digest newly infected DNA [55]. The result is targeted cutting of double stranded DNA that is specific and highly efficient. Multiple groups have adapted this technology to mammalian systems to improve upon preexisting methodologies and have confirmed that this tool is highly accessible and amenable to targeted genome editing [56]. Despite the promise of this technology, there remains a paucity of studies that examine the AHR within the context of iPSC directed differentiation [25, 57] or incorporate CRISPR/CAS9 to employ genetic manipulation of AHR signaling [58]. Future work will help reveal the signaling dynamics and interregulatory cross talk of the AHR pathway in differentiated iPSCs within multiple cellular contexts.

Using a CRISPR/CAS9 system for genome editing, we have created an endogenous reporter of AHR activity in an iPSC line by targeting the* CYP1A1* locus.* CYP1A1* is a canonical target of AHR signaling, one that is widely used to report on AHR activity in multiple cell and tissue types* in vivo* and* in vitro*. As a result, its expression is commonly used as a functional output of AHR activity in the absence of ligand exposure. In this report, we show the utility of this cell line in its response to multiple agonists and antagonists and validate its function by observing AHR modulation in the context of both hematopoiesis and hepatic specification.

## 2. Materials and Methods

### 2.1. iPSC Generation and Maintenance

Induced pluripotent stem cells were generated as described previously [59, 60]. Briefly, 4 mL of human peripheral blood was collected into a BD Vacutainer CPT Cell Preparation Tube and centrifuged to produce a buffy coat containing peripheral blood mononuclear cells (PBMCs). The buffy coat was collected and PBMCs were cultured* ex vivo* for 9 days before being transduced with the STEMCCA lentiviral vector. At day 12 of culture, STEMCCA transduced PBMCs were plated onto mouse embryonic fibroblasts (MEFs) and cultured until roughly days 30–40, when fully formed iPSC colonies were identified and separately harvested. Following successive passages onto irradiated MEFs (R&D, #PSC001), colonies were adapted to matrigel-coated tissue culture dishes in the absence of a feeder cell layer. iPSCs were then cultured in mTESR1 media (StemCell Technologies, #05850) for all further passages.

### 2.2. Creation of CRISPR/CAS9 Targeted* CYP1A1* Reporter iPSCs

**Figure 1:**
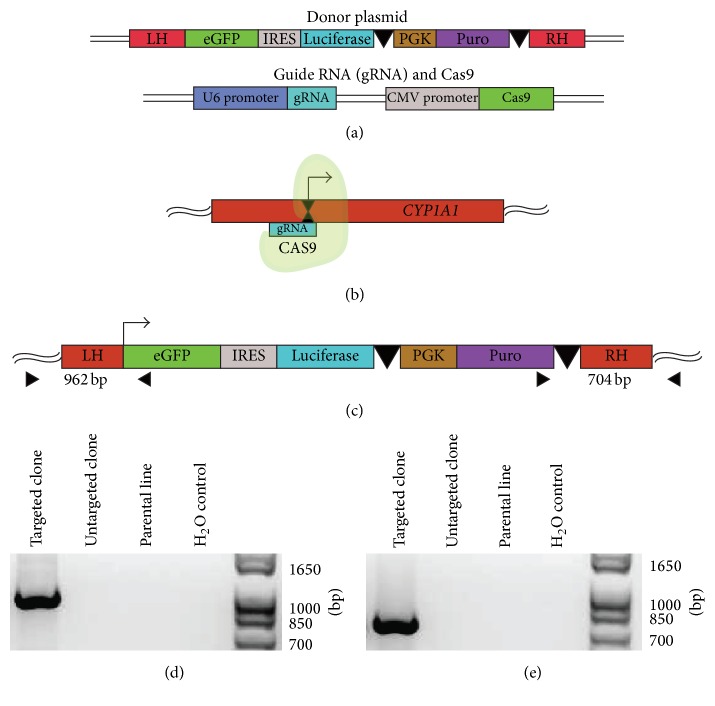
*Construction and validation of CYP1A1 reporter iPSCs.* (a) Two vectors were created to achieve CAS9 targeted digestion at the* CYP1A1 *transcription start site and homologous recombination of a reporter construct. The donor plasmid contains a cassette that includes eGFP and luciferase separated by an internal ribosome entry sequence (IRES). Directly downstream of these reporter elements is a puromycin resistance gene (Puro) driven by a constitutive promoter (PGK) and flanked by loxP sites (denoted by black arrowheads). This cassette is flanked by regions that are homologous to the* CYP1A1* endogenous locus (Left Homology, LH; Right Homology, RH) to facilitate homologous recombination. The guide RNA (gRNA) and Cas9 were encoded on the same plasmid, each driven by a separate constitutive promoter (U6 and CMV, resp.). (b) An idealized schematic of Cas9 digestion at the transcription start site (denoted by black arrow) of the* CYP1A1* locus. (c) The integrated reporter construct is expected to specifically target the transcription start site of* CYP1A1*, and a PCR strategy was employed that creates amplicons in the 5′ and 3′ flanking regions of the cassette that include elements from the reporter construct as well as endogenous regions that are not encoded by the donor plasmid. (d) The 5′ amplicon (expected size = 962 bp) was exclusively detected in a properly targeted iPSC clone. (e) The 3′ amplicon (expected size = 704 bp) also could not be amplified in untargeted clones or the parental iPSC line but was detected in a properly targeted iPSC clone.

Targeting of the* CYP1A1* locus was achieved by cotransfection of the plasmids described (Figure 1(a)). Confluent iPSC cultures were pretreated with 10 *μ*M Y-27632 (ROCK inhibitor) for 3 hours in mTESR1 medium. Cells were resuspended in 100 *μ*L of P3 solution (Lonza) and added to a cuvette for the Lonza 4D Nucleofector at a density of 5e6 cells per cuvette. 2 *μ*g of the CAS9 vector and 3 *μ*g of the Donor vector were added to the cell suspension and nucleofected using the CB-150 program. Immediately following nucleofection, cells were resuspended into fresh mTESR1 with 10 *μ*M Y-27632 and plated onto one 10 cm plate (pretreated with matrigel) and left at 37° in a low oxygen (5% O_2_) incubator. Cells were allowed to grow for 5 days before clones were selected for puromycin resistance by the addition of 0.7 *μ*g/mL puromycin (ThermoFisher, #A1113802). Colonies were harvested as they appeared in culture and were passaged and maintained separately before being screened by PCR for the integrated construct.

### 2.3. PCR and Sanger Sequencing

To validate proper targeting of the reporter construct to the* CYP1A1* locus, two PCR products were amplified that flank the 5′ and 3′ ends of the reporter construct (resp.) and include elements of both the integrated cassette and the endogenous locus (Figure 1(c)). PCR was performed using recombinant Taq polymerase (ThermoFisher, #10342) with primers for the 5′ amplicon (Forward: 5′-ggtgggatttcctgcatcct-3′; Reverse: 5′-cttgtggccgtttacgtcg-3′) and 3′ amplicon (Forward: 5′-cctgcaggatctgatcagataacttcg-3′; Reverse: 5′-caggttgactaggctaagcagttcttg-3′) in separate reactions. PCR products were resolved by agarose gel electrophoresis and were 962 bp and 704 bp, respectively. Bands that appeared to be the proper size by electrophoresis were extracted and purified using the QiaQuick gel extraction kit (Qiagen, #28704) and submitted for Sanger sequencing to Genewiz, Inc. Sequencing data, as well as all homology domain and guide RNA sequences, are available in Supplemental Figure  1 in Supplementary Material available online at http://dx.doi.org/10.1155/2016/2574152.

### 2.4. Generation of Hepatocyte-Like Cells from iPSCs

iPSC cultures were passaged using Gentle Cell Dissociation (GCD) Reagent (StemCell Technologies, #07174) to obtain a single cell suspension and counted using a hemacytometer. Cells were passaged onto matrigel-coated tissue culture dishes at a cellular density of 3e5 per well of a standard 6-well plate. After 24 hours, mTESR1 was replaced by media provided by the STEMdiff Definitive Endoderm Kit (StemCell Technologies, #05110) and cultured according to manufacturer's instructions for 5 days. At day 5, GCD was used to make a single cell suspension and the cells were passaged at a ratio of 1 : 6 onto matrigel-coated 6 well plates. The media for all subsequent days were an SFD base [61] with ascorbic acid (50 *μ*g/mL) and monothioglycerol (4.5*e* − 4 M). Media for days 5 and 6 included Activin A (50 ng/mL), BMP4 (10 ng/mL), FGF2 (10 ng/mL), and VEGF (10 ng/mL). The media for days 7–12, days 13–18, and days 19–25 were adapted directly from a previous manuscript [35]: days 7–12: BMP4 (50 ng/mL), FGF2 (10 ng/mL), VEGF (10 ng/mL), EGF (10 ng/mL), TGFa (20 ng/mL), HGF (100 ng/mL), and 0.1 *μ*M Dexamethasone; days 13–18: FGF2 (10 ng/mL), VEGF (10 ng/mL), EGF (10 ng/mL), HGF (100 ng/mL), Oncostatin M (20 ng/mL), Vitamin K (6 *μ*g/mL), 1.5 *μ*M gamma secretase inhibitor, 0.1 *μ*M Dexamethasone, and 1% DMSO; days 19–25: HGF (100 ng/mL), Oncostatin M (20 ng/mL), Vitamin K (6 *μ*g/mL), and 0.1 *μ*M Dexamethasone. Cells were kept in a low oxygen (5% O_2_) incubator throughout the differentiation.

### 2.5. Generation of Hematopoietic Progenitor Cells from iPSCs and Treatment with 6-Formylindolo(3,2-b)carbazole (FICZ)

Hematopoietic progenitor cells were derived from induced pluripotent stem cells using our previously published protocol [25]. Briefly, iPSCs seeded on matrigel plates were exposed to cytokine conditions that promoted mesoderm specification, followed by a hemogenic endothelial-like phenotype, and, finally, hematopoietic progenitors that disadhered from the matrigel substrate and were dual positive for CD41 and CD235 (data not shown). At day 7 of the protocol (Supplemental Figure  2), cells were treated with escalating doses of FICZ, at a range of 10*e* − 8 to 10*e* − 4 M, and kept in this condition for 5 days. Cells were harvested at day 12, at which point lysates were created for luciferase assays as well as RNA extraction and kept at −80°C.

### 2.6. RNA Extraction and Quantitative PCR

RNA was extracted using the RNeasy Mini Kit (Qiagen, #74104). At the time of harvest, cells were washed with PBS and spun for 5 minutes at 300 ×g, and the pellet was collected in 350 *μ*L of Buffer RLT. RNA extraction proceeded according to manufacturer instructions. RNA was eluted into 30 *μ*L of endonuclease-free H_2_O and purified with DNAse using the DNA-free DNA Removal kit (ThermoFisher, #AM1906). Once purified, 20 *μ*L of sample was used to generate cDNA using the High-Capacity cDNA Reverse Transcription Kit (Applied Biosystems, #4368814). RNA samples were quantified using a NanoDrop Lite (Thermo Scientific) Spectrophotometer, and cDNA samples were diluted to 1 *μ*g/*μ*L. Quantitative PCR was carried out using the Taqman Universal Master Mix (Thermo Scientific) and primers for* CYP1A1* (Hs01054797_g1) and *β-ACTIN* (Hs99999903_m1) were used. Samples were run in triplicate and, where appropriate, were analyzed by Student's *t*-test to assess significance between groups.

### 2.7. Flow Cytometry

Flow cytometry was performed at days 5, 14, and 25 of hepatocyte specification. For day 5, 3e5 cells were stained per condition, and the C-KIT antibody (Biolegend, #313206) and CXCR4 antibody (Invitrogen, #MHCXCR404) were used at a concentration of 5 *μ*L per 1e6 cells. Staining was performed on ice for 30 minutes. For days 14 and 25, cells were fixed in 1.6% paraformaldehyde before staining. Primary antibodies for AAT (Santa Cruz, #sc-59438) and FOXA1 (Santa Cruz, #101058) were added at 1 : 100 ratio in Saponin Buffer (2% FBS, 1x Permeabilization Wash Buffer (Biolegend, #421002)) and incubated at room temperature for 30 minutes. Secondary antibodies for AAT (Jackson Immunoresearch, #115-605-205) and FOXA1 (Jackson Immunoresearch, #115-545-206) were added at a dilution of 1 : 500 and incubated at room temperature for 30 minutes. All samples were resuspended in PBS with 0.5% BSA for analysis.

### 2.8. Luciferase Assays

To assess luciferase expression, cells were harvested and counted by hemacytometer to ensure 1e5 cells per 20 *μ*L of 1x lysis buffer from the commercially available luciferase assay system (Promega, #E1500). Upon sufficient lysis, samples were stored at −80°C until all time points were collected. Samples were then thawed on ice and assayed by adding 20 *μ*L per well of a 96-well plate, followed by addition of 100 *μ*L of luciferase assay reagent (Promega) and immediate analysis of luminescence in a Tecan Infinite M1000 microplate reader.

### 2.9. Fibroblast Differentiation and Small Molecule Treatment

iPSCs were seeded on matrigel-coated 12-well plates at a density of 3e5 cells per well and left in mTESR medium for 24 hours. Fibroblast induction media (IMDM, 10% FBS, 2 mM l-glutamine, and 100 *μ*g/mL primocin (Invivogen, #ant-pm-1)) were added for 2 days, followed by small molecule treatment for exactly 24 hours before cells were harvested for luciferase and qPCR assays. Small molecule AHR modulators were added at the following concentrations: TCDD, 1 nM; CH223191, 10 *μ*M; benzo[a]pyrene, 1 *μ*M; benzo[e]pyrene, 1 *μ*M; FICZ, 10 *μ*M; indoxyl sulfate, 100 *μ*M.

### 2.10. Image Capture and Analysis

All images were captured on a Nikon Eclipse TS100 microscope equipped with a Diagnostic Instruments, Inc., model 18.2 Color Masonic Camera. Images were processed using Adobe Illustrator software.

### 2.11. Statistical Analysis

Results are presented as mean ± the standard error of the mean (SEM). Statistical significance was confirmed using the Student *t*-test as indicated.

## 3. Results and Discussion

### 3.1. Vector Design and Construction and Validation of a* CYP1A1* Reporter iPSC Line

To create an endogenous reporter of AHR activity in an iPSC line, a CRISPR/CAS9 system was engineered to target the* CYP1A1* locus. A plasmid was created that expresses a reporter cassette of enhanced green fluorescent protein (eGFP) and firefly luciferase bifurcated by an internal ribosomal entry sequence (IRES) to allow for each reporter gene to be expressed on the same transcript (Figure 1(a)). Downstream of this cassette is a puromycin resistance gene (PURO) driven by a constitutive promoter for murine phosphoglycerate kinase (PGK) to allow for antibiotic selection. This reporter sequence does not include a 5′ regulatory region but rather is flanked by homology arms that facilitate recombination directly downstream of the* CYP1A1* transcription start site in the endogenous locus (Supplemental Figure  1B). Using this strategy, reporter expression is exclusively driven by the* CYP1A1* promoter, a regulatory region that includes 10 distinct AHR response elements (AHREs) [62]. A guide RNA sequence (gRNA) was developed using a publically available web resource (http://crispr.mit.edu/) created and distributed by the Zhang Lab at the Massachusetts Institute of Technology [63]. The guide RNA shares sequence homology with a 23-base-pair region exactly 8 base pairs downstream of the* CYP1A1* start codon (5′-CCCAATCTCCATGTCGGCCACGG-3′) that includes a 3-base-pair protospacer adjacent motif (PAM) sequence that is necessary for CAS9 binding (Figure 1(b); Supplemental Figure  1B). The guide RNA and CAS9 coding regions were included on the same plasmid, separate from the plasmid containing the reporter construct, each with a dedicated constitutive promoter (Figure 1(a)).

Cotransfection of the two engineered plasmids produced a series of puromycin resistant clones which were then screened for the inserted reporter sequence within the* CYP1A1* locus. Validation was accomplished by a PCR strategy that creates two distinct amplicons at the flanking regions of the integrated cassette. Using this strategy, each amplified region contains elements of the* CYP1A1* endogenous locus that is not included in the homology arms as well as elements of the donor sequence (Figure 1(c)). Successful PCR amplification of these regions can only be achieved in properly targeted clones (Figures 1(d) and 1(e)) and Sanger sequencing confirms that each amplicon includes genomic regions of the* CYP1A1* locus as well as elements from the donor construct (Supplemental Figure  1A).

### 3.2. *CYP1A1* Reporter iPSCs Respond to FICZ in a Dose-Dependent Manner

To achieve functional validation of the properly targeted clone, we used a previously published, directed differentiation protocol for the production of hematopoietic progenitors of the megakaryocyte and erythroid lineages [25]. Our previous work revealed that activation of the AHR pathway with 6-formylindolo(3,2-b)carbazole (FICZ) in this population causes exponential expansion and increased viability in culture [25]. Having proven this population's responsiveness to AHR agonism, we treated hematopoietic progenitors derived from a* CYP1A1* targeted clone with escalating doses of FICZ for 5 days. FICZ treatment increased transcript expression of* CYP1A1* in a dose-dependent manner, and this result was observed in both the parental iPSC line and the* CYP1A1* targeted clone (Supplemental Figure  2A). These cultures were also assayed for luciferase expression, and unlike* CYP1A1* transcript expression, only the* CYP1A1* targeted line displayed luciferase bioluminescence that increased significantly with each successive FICZ dose (Supplemental Figure  2B). This work confirms that the CRISPR/CAS9 targeted clone faithfully reports on AHR activation through a functional output of luciferase expression.

### 3.3. Mapping of AHR Activity throughout Human Hepatocyte Specification Using* CYP1A1* Targeted iPSCs

**Figure 2:**
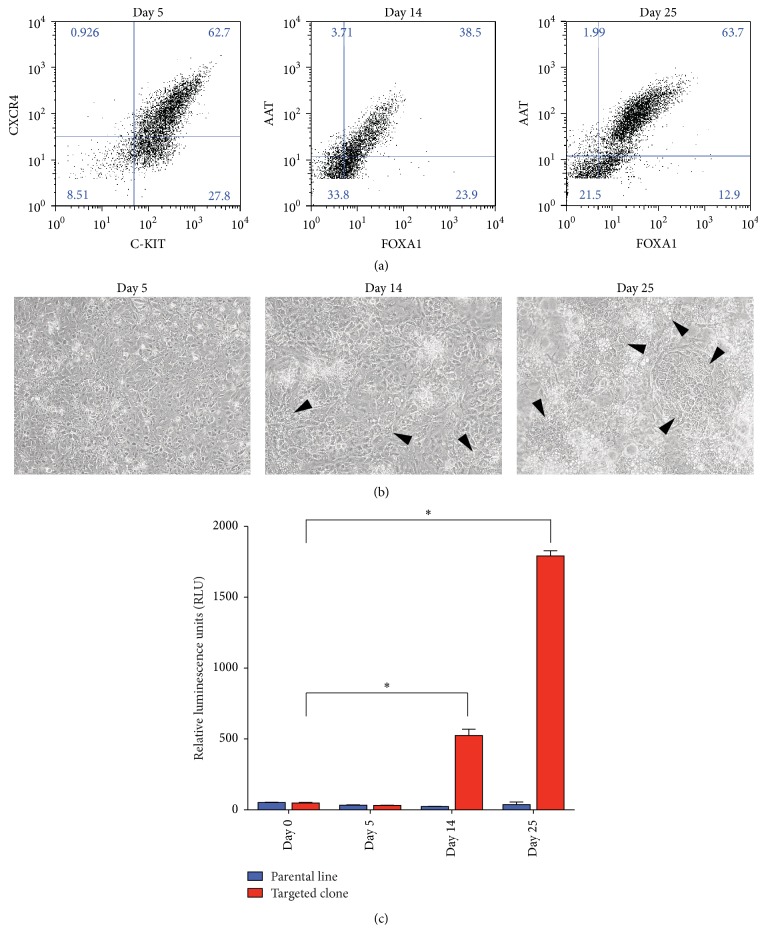
Hepatocyte specification yields luciferase expression in cells derived from* CYP1A1* reporter iPSCs. (a) iPSCs were differentiated towards CXCR4+/C-KIT+ definitive endoderm (day 5) followed by FOXA1+/AAT+ hepatic progenitors (day 14) that grew in number and were the majority of the culture by day 25. (b) Micrographs show homogenous morphology of definitive endoderm cultures (day 5), but by day 14, hepatic-like cells begin to emerge (denoted by black arrowheads) and are observed more frequently by day 25. (c) Concomitant with hepatic specification, luciferase levels significantly increase (*N* = 3, ^*∗*^ *P* < 0.0005, Student's *t*-test).

To fully utilize the* CYP1A1* targeted iPSC line, we differentiated these cells towards the hepatocyte lineage in order to showcase the potential of this reagent to provide a temporal map of AHR activation in a variety of cellular contexts. Multiple studies have reported on the AHR response to environmental ligands in primary liver and have displayed a baseline level of* CYP1A1* expression even in the absence of toxin exposure [64]. Thus, in order to confirm the utility of this cell line, we sought to recapitulate these results in an* in vitro* context using a previously described protocol for directed differentiation to hepatocyte specification [35, 36]. Using this strategy, we successfully produced cells with definitive endodermal markers (CXCR4 and CKIT) after 5 days of differentiation and proceeded to incorporate a cytokine cocktail including FGF2 and Activin A to produce early hepatocyte progenitors at day 14, as indicated by observed dual positivity for Alpha 1 Antitrypsin (AAT) and FoxA1 (Figure 2(a)). Cultures were subsequently exposed to a specified media containing Hepatic Growth Factor (HGF) and Oncostatin M, and at day 25, the AAT+/FoxA1+ population had increased substantially (63.7%) (Figure 2(a)). Micrographs taken at days 5, 14, and 25 of differentiation show the progressive change in cellular morphology of these cells as they formed a homogenous 2D monolayer (day 5) followed by a heterogenous population where polygonal hepatic-like cells began to emerge (day 14) and, finally, an adherent cellular layer dominated by granular, polygonal cells with distinct, sinusoidal-like boundaries (day 25) (Figure 2(b)). The* CYP1A1* reporter clone was differentiated in parallel with the parental iPSC line, and luciferase-dependent bioluminescence was assayed at each time point throughout the hepatic differentiation. Undifferentiated cells (day 0) as well as CXCR4+/CKIT+ definitive endoderm (day 5) produced low levels of* CYP1A1*-driven luciferase, but a marked increase in luciferase expression was observed in hepatocyte progenitors (day 14) and, more significantly, early hepatocytes (day 25) (Figure 2(c)). Interestingly, the discrepancy seen between these two final time points is highly correlated to the relative abundance of AAT+/FoxA1+ dual positive cells within these cultures, suggesting that AHR activation occurs exclusively in this discrete population. The ability of* CYP1A1* targeted iPSCs to faithfully report on patterns of activation previously reported in primary cells is an early indication of the utility of this cell line in mimicking* in vivo* ontogeny and providing an easily accessible model system upon which to study this highly ubiquitous pathway.

### 3.4. *CYP1A1* Reporter iPSC-Derived Fibroblast-Like Cells Respond to Putative AHR Ligands

**Figure 3:**
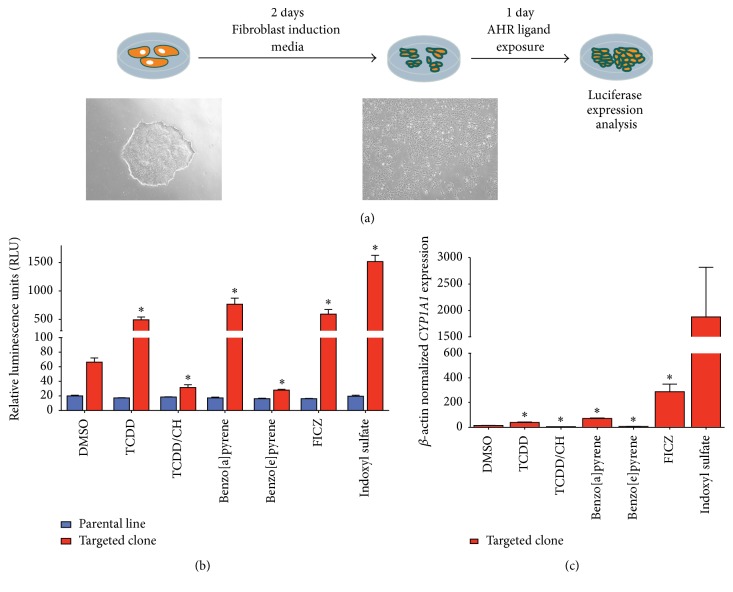
Acute exposure to AHR ligands causes a predictable response in* CYP1A1* reporter iPSCs. (a) Fibroblast-like cells were differentiated from* CYP1A1* iPSCs over the course of 2 days using fibroblast induction media. A compendium of AHR ligands were then added to the cultures for 24 hours before cells were harvested for further analysis. (b) Luciferase expression analysis reveals patterns of activation and inhibition of the* CYP1A1* reporter (targeted clone) as a response to agonist and antagonist treatment. Significance was established by Student's *t*-test for each condition compared to the DMSO control condition (^*∗*^ *P* < 0.01, *N* = 3) (c) Transcript level expression of endogenous* CYP1A1* in the targeted clone showed similar patterns of modulated expression that positively correlated to luciferase output. Significance was established by Student's *t*-test for each condition compared to the DMSO control condition (^*∗*^ *P* < 0.02, *N* = 3).

With the evolution of the AHR field, culminating in the description of endogenous roles of the AHR in the absence of classical, environmentally derived ligands, the value of an iPSC clone with the capacity to report on AHR activation is dependent upon its sensitivity to multiple small molecule compounds previously shown to affect AHR signaling [65–67]. To assess the ability of the* CYP1A1* targeted cell line to respond to exogenous and proposed endogenous AHR ligands, we ran a comprehensive chemical screen and assayed for luciferase-dependent luminescence at 24 hours after dosing. Due to the observed lack of AHR dependent luciferase expression in the undifferentiated state (Figure 2(c)) we exposed iPSCs to a simplified media over the course of two days that quickly altered the cellular morphology to a fibroblast-like appearance (Figure 3(a)). These cells had detectable luminescence in the naïve (untreated) condition (Figure 3(b)), whereas undifferentiated cells had an indistinguishable expression profile to that of the parental iPSC line (Figure 2(c);* day 0 time point*). Our chemical screen incorporated known environmental ligands, including TCDD and benzo[a]pyrene, as well as endogenous agonists' indoxyl sulfate and FICZ, and a potent AHR antagonist, CH223191 (CH). Treatment with DMSO alone established the basal levels of luciferase output, and TCDD induced a significant response that was completely occluded by the presence of CH223191 (Figure 3(b);* TCDD/CH condition*). Both of these compounds are known to affect gene expression in an AHR dependent fashion, giving further credence to the specificity of this AHR reporter system. Further, the polycyclic aromatic hydrocarbon benzo[a]pyrene induced AHR activity while benzo[e]pyrene, a structurally similar compound previously shown to have very little affinity for AHR in the cytoplasm [68], actually seemed to inhibit activity relative to the vehicle control. Additionally, the tryptophan derivatives indoxyl sulfate and 6-formylindolo(3,2-b)carbazole (FICZ) also proved efficacious in this model system. Finally,* CYP1A1* gene expression was assessed and found to be modulated in a similar pattern to that of luciferase expression (Figure 3(c)), further substantiating the hypothesis that luciferase expression reports directly on AHR activation.

## 4. Conclusions

Multiple bioassays have been developed to study AHR signaling in distinct cellular subtypes. These systems' utility has mainly been in the identification of environmental ligands [69] and, recently, the discovery of proposed endogenous ligands that range from the tryptophan derivates FICZ [70, 71] and indoxyl sulfate [72] to bilirubin, a natural product of heme catabolism [73, 74] and the arachidonic acid metabolites prostaglandin G [75] and lipoxin A4 [76]. Perhaps the most widely utilized experimental reporter is the pGudLuc vector, of which multiple iterations have been reported in the literature [69]. This vector was created by incorporating a 482-base-pair segment of the murine* CYP1A1* promoter with 4 AHREs (also known as Dioxin Response Elements, or DREs) within the mouse mammary tumor virus (MMTV) promoter, with luciferase expression as the functional readout. Since its inception [77], this reagent has been optimized for better stability [69] and used by many groups to assess ligand responsiveness in immortalized cell models [78, 79].

The endogenous reporter of AHR activity described in this work represents a significant technological advancement that is highly specific and accessible to researchers with various expertise. Unlike immortalized cell lines with stable transfection of pGudLuc, this iPSC line has a targeted integrant directly downstream of the* CYP1A1* transcription start site. Despite observations in the literature of off-target CRISPR/CAS9 cutting [80], the functional data presented herein substantiates our hypothesis that little to no off-target integration of the reporter construct exists in this particular case. We hypothesize, then, that this reporter line is isogenic to an in-house iPSC control and can be efficiently differentiated to multiple cellular lineages. Transcript level expression of* CYP1A1* in the targeted line upon FICZ treatment (Supplemental Figure  2) proves that endogenous* CYP1A1* is not knocked-out as a result of genomic integration, making it likely that the reporter construct is hemizygously expressed. This would indicate that this clone has a single integrant in the exact genomic location that AHR:ARNT dimers naturally modulate* CYP1A1* gene expression. This system avoids random integration of an artificial promoter driven construct that could be expressed in multiple genomic locations, affecting endogenous gene expression in unknown ways. It also uses the entire* CYP1A1* promoter to drive expression, utilizing potentially complex interactions and gene expression profiles dependent on distal cis elements that cannot be conveyed by transfection of reporter plasmids. TCDD exposure, for example, is known to affect local chromatin structure in promoting endogenous* CYP1A1* expression [81] and selective ligands may alter AHR:ARNT dimer binding, causing AHRE-independent control of the* CYP1A1* promoter [82]. Indeed, our finding that benzo[e]pyrene inhibits* CYP1A1* expression in fibroblast-like cells (Figure 3) contradicts reports of its lack of affinity towards the AHR and warrants further investigation. Thus, this cell line will be critically important in future studies that implicate novel small molecule compounds as AHR modulators.

iPSC technology continues to be an attractive avenue for basic science to achieve clinically relevant applications. Less than a decade after their inception, iPSCs are being used as a source of cellular therapeutics [82] and have undergone successful gene correction in lines created from primary cells of patient populations [83]. Now, there is the potential to turn iPSC-derived cultures into drug screening tools that can provide early indices of safety and efficacy before patient populations are exposed [84–87]. Given the widely reported role of AHR signaling in hepatotoxicity [88] and carcinogenesis [89], our reporter iPSC line is an optimal tool to reveal potential toxicity of compounds of interest in preclinical phases of development.

As the full extent of AHR pathway dynamics is discovered and the mechanisms of endogenous ligand regulation dominate the literature, it will become paramount to map AHR activation throughout all phases of development. Induced pluripotent stem cells provide an invaluable tool by which to derive distinct cellular subtypes of all three germ layers, and the* CYP1A1* reporter line presented in this work can provide an output of AHR activity that can be observed in every experimental context. Differentiation strategies that mimic* in vivo* ontogeny have the potential to serve as “temporal maps” of AHR activity throughout cytokine driven progression of cells to a distinct lineage. To this end, cellular fate decisions of progenitor populations can be correlated to AHR expression, and terminally differentiated cells can be assayed for AHR activity relative to their specification and function. Multiple novel roles of AHR signaling as well as the identity and dynamics of endogenous ligands have yet to be discovered; the reporter iPSC line described in this work will be invaluable to these studies moving forward.

## Supplementary Material

Supplemental Figure 1: Sanger sequencing confirms PCR validation strategy of *CYP1A1* reporter iPSCs. (A) PCR products discussed in Figure 1C were purified and sequenced using the Sanger method (Genewiz, Inc.). Both amplicons include endogenous regions of the *CYP1A1* locus as well as elements of the reporter construct (eGFP in the 5′ amplicon; puro resistance gene in the 3′ amplicon). (B) Full sequences for the Left Homology Arm, Right Homology Arm, and single guide RNA (sgRNA).
Supplemental Figure 2: *CYP1A1* reporter iPSCs elicit a response to the AHR agonist FICZ. (A) *CYP1A1* transcript level expression was increased in both the parental cell line (blue bar) and the targeted clone (red bar) in response to escalating doses of 6-formylindolo(3,2-b)carbazole (FICZ). (B) Luciferase expression was observed as a result of FICZ treatment exclusively in the targeted clone (red bar) but not in the parental cell line (blue bar), showing that the integrated reporter construct was faithfully reporting on AHR-dependent *CYP1A1* upregulation.
