# Supplementary material for: Genome Editing of the CYP1A1 Locus in iPSCs as a Platform to Map AHR Expression throughout Human Development
Source: Stem Cells Int. 2016 Apr 11;2016:2574152. doi: 10.1155/2016/2574152 (PMC4842384; doi:10.1155/2016/2574152)
Supplement: Supplementary file 1 — Supplemental Figure 1: Sanger sequencing confirms PCR validation strategy of CYP1A1 reporter iPSCs. (A) PCR products discussed in Figure 1C were purified and sequenced using the Sanger method (Genewiz, Inc.). Both amplicons include endogenous regions of the CYP1A1 locus as well as elements of the reporter construct (eGFP in the 5′ amplicon; puro resistance gene in the 3′ amplicon). (B) Full sequences for the Left Homology Arm, Right Homology Arm, and single guide RNA (sgRNA). Supplemental Figure 2: CYP1A1 reporter iPSCs elicit a response to the AHR agonist FICZ. (A) CYP1A1 transcript level expression was increased in both the parental cell line (blue bar) and the targeted clone (red bar) in response to escalating doses of 6-formylindolo(3,2-b)carbazole (FICZ). (B) Luciferase expression was observed as a result of FICZ treatment exclusively in the targeted clone (red bar) but not in the parental cell line (blue bar), showing that the integrated reporter construct was faithfully reporting on AHR-dependent CYP1A1 upregulation. [file 2574152.f1.pdf]

A

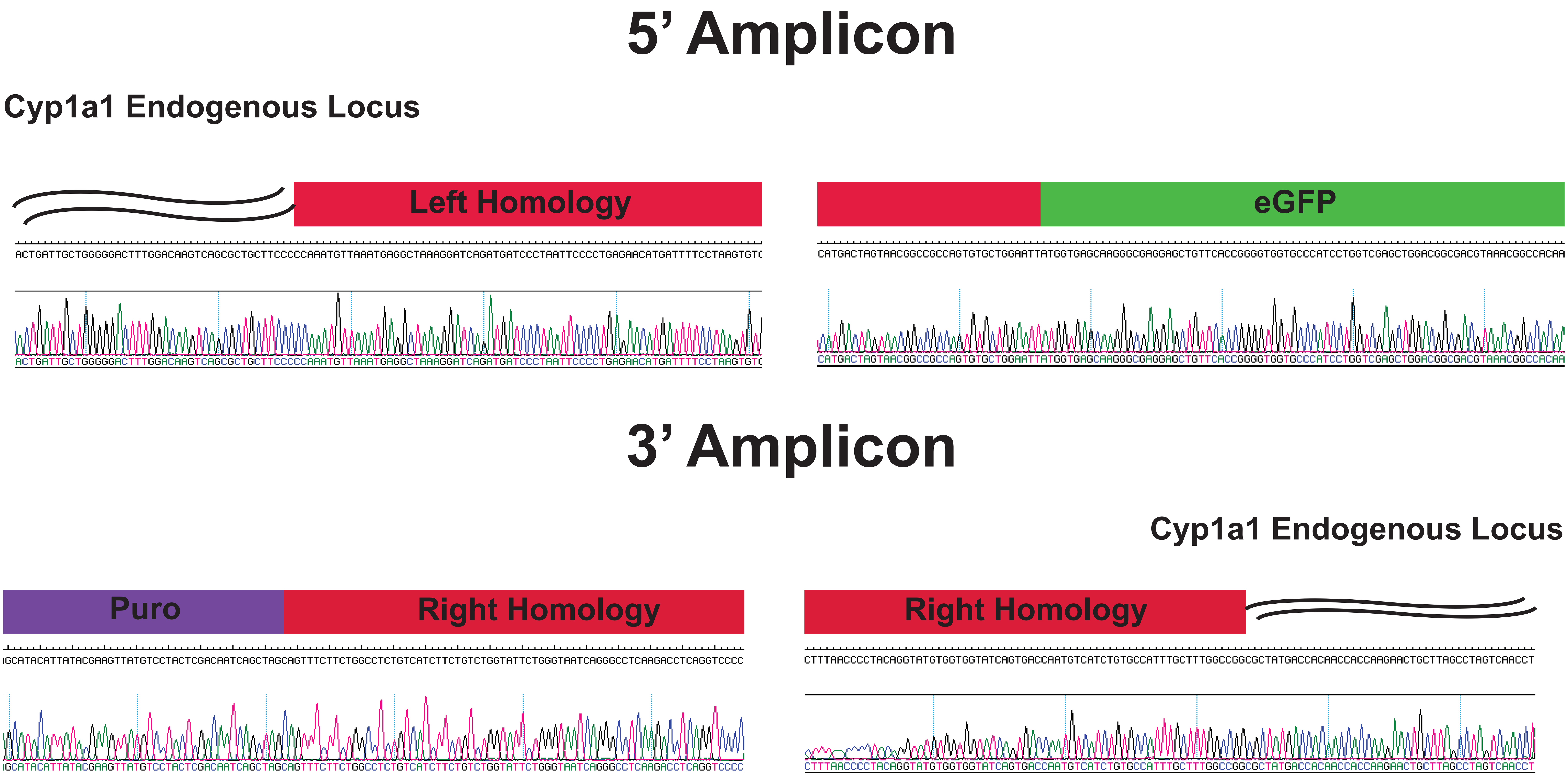

B

Left Homology Domain:
5′-ccaaatgttaaatgaggctaaaggatcagatgatccctaattcccctgagaacatgattttcctaagtgtggtgggtaggactcggggtgatacaccttgattatgtgtttattttaatgtgttaggggaaaaatcaaacttgtactttcac atagatattgtttatgatgatatgtaggtagatttaaagttgattttaggaaaatgtgaagtaaataacagtactggtactatgtggaaatcacagattgacattggaagatgtcagtttagattggtacagcagttaagagaaagcgttctg gaattaggcaacttggcttgggagtctgcatccaacactttctggctgtgtgaccttgggcaagttacttaccttctctgagcctcattgcaggctacttcatagatttgtggccagggctgaatgaagaaggactgtgaaattcttagccca cagtggtagttcaacaacttctccccacctcaccctcttcttcatgcccagtaagcagttctggtggaaaggtttcccctttccctgacactctagatattggcttttctcatcccccaatctgacggcttgactttttttcttcctgcaccttctctca gcagccacctccaagatccctacactgatca**-3′**

Right Homology Domain:
5′-agtttcttctggcctctgtcatcttctgtctggtattctgggtaatcagggcctcaagacctcaggtccccaaaggcctgaagaatccaccagggccatggggctggcctctgattgggcacatgctgaccctgggaaagaacccgca cctggcactgtcaaggatgagccagcagtatggggacgtgctgcagatccgaattggctccacacccgtggtggtgctgagcggcctggacaccatccggcaggccctggtgcggcagggcgatgatttcaagggccggcccgac ctctacaccttcaccctcatcagtaatggtcagagcatgtccttcagcccagactctggaccagtgtgggctgcccgccggcgcctggcccagaatggcctgaaaagtttctccattgcctctgacccagcctcctcaacctcctgctacctg gaagagcatgtgagcaaggaggctgaggtcctgataagcacgttgcaggagctgatggcagggcctgggcactttaacccctacaggtatgtggtggtatcagtgaccaatgtcatctgtgccatttgctttggccgg**-3′**

Single Guide RNA (sgRNA):
5′-cccaatctccatgtcggccacgg**-3′**

Supplemental Figure 1: Sanger sequencing confirms PCR validation strategy of *CYP1A1* reporter iPSCs A) PCR products discussed in Figure 1C were purified and sequenced using the Sanger method (Genewiz, Inc.). Both amplicons include endogenous regions of the *CYP1A1* locus as well as elements of the reporter construct (eGFP in the 5' amplicon; puro resistance gene in the 3' amplicon). B) Full sequences for the Left Homology Arm, Right Homology Arm, and single guide RNA (sgRNA).

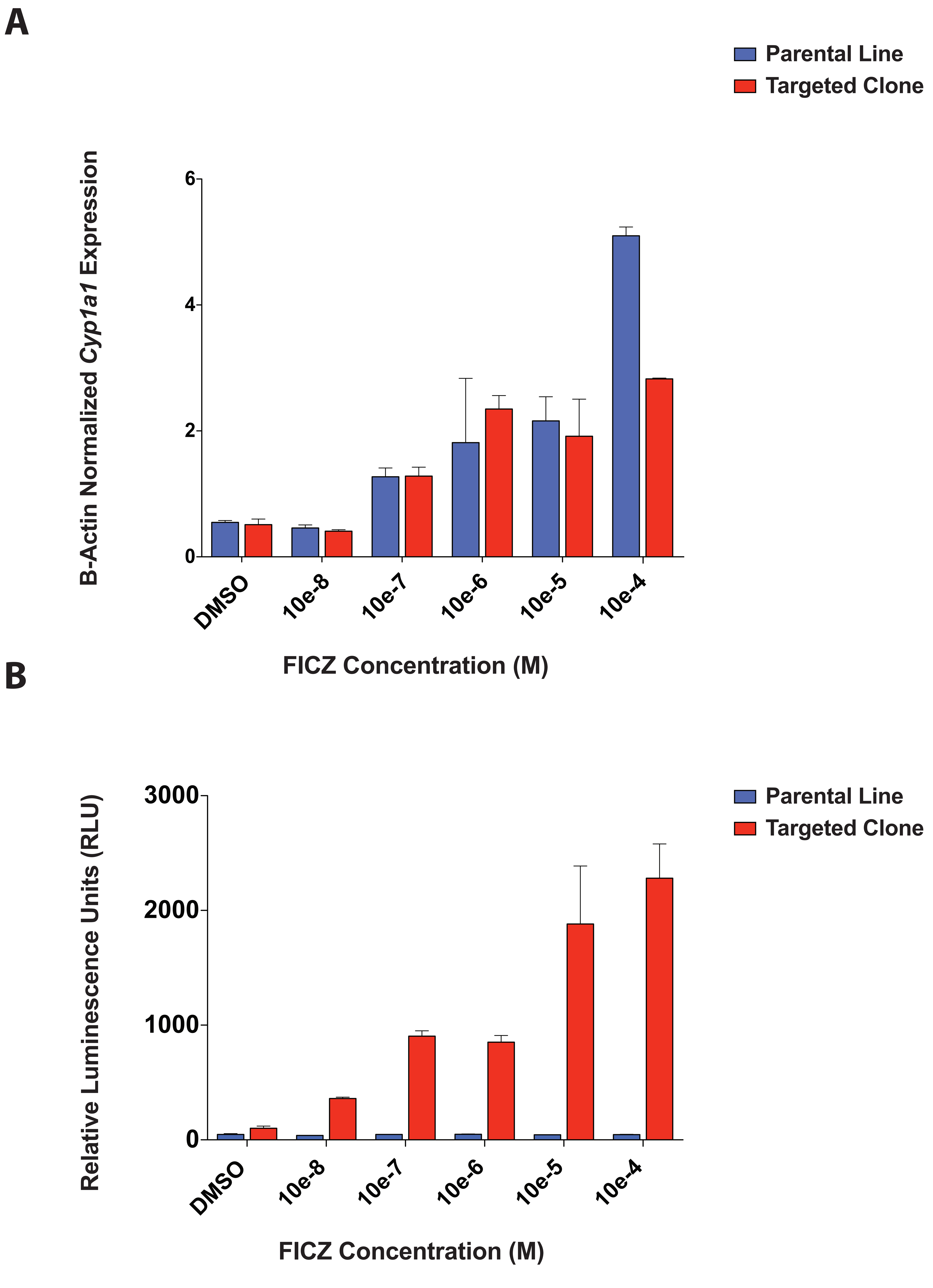

Supplemental Figure 2: *CYP1A1* reporter iPSCs elicit a response to the AHR agonist FICZ A) *CYP1A1* transcript level expression was increased in both the parental cell line (blue bar) and the targeted clone (red bar) in response to escalating doses of 6-formylindolo(3,2-b)carbazole (FICZ). B) Luciferase expression was observed as a result of FICZ treatment exclusively in the targeted clone (red bar) but not in the parental cell line (blue bar), showing that the integrated reporter construct was faithfully reporting on AHR-dependent *CYP1A1* upregulation.
